# New York Dental Recorder

**Published:** 1848-10

**Authors:** 


### NEW YORK DENTAL RECORDER.

In the June No. of our valued cotemporary, the New York Dental Recorder, some exception is taken by its Editor to certain "Comments" which we felt it our duty to make on the "Actions of the American Society of Dental Surgeons." Our remarks were intended for those who merited their application; they were aimed at no particular person, and as we entertain no ill-will towards any man, it cannot be supposed that we would, intentionally, wound the feelings of others; we may also add, that our only means of learning what the Society had done, was through the published record, contained in the "American Journal of Dental Science." As we have no where seen its correctness questioned, until we received the No. referred to, no blame can possibly attach to us for throwing our *ink* somewhat indiscriminately in the premises.

It is notso *easy* a matter, as it is intimated, to travel from Saint Louis is to New York, a distance of fifteen or eighteen hundred miles, to at tend the annual meetings of the Society,—not to mention the small matter of *expense*. On one occasion we made the effort for the sole purpose of being present at its deliberations, and unfortunately for us, we remained long enough on a sand-bar in the Ohio river to deprive us of the pleasure of meeting with the Am. Soc. of Dent. Surgeons, so that when we arrived in the East, we were "just *soon enough to be too late*," the Society having adjourned several days previously. Before closing, we must congratulate our talented cotemporary for having passed through the campaign so successfully, and must beg leave to thank him for the humanity and respect which he has exhibited towards the *dead*, and the unremitting attention which he has gallantly given to the *wounded*. We hope he has ultimately succeeded in coming off much better than the Texian did in the Bear-fight; he was perfectly satisfied to get off *minus* every thing but his *shirt-collar* and *one spur*.—St. Louis Ed.

